# Demographic and Spatiotemporal Patterns of Avian Influenza Infection at the Continental Scale, and in Relation to Annual Life Cycle of a Migratory Host

**DOI:** 10.1371/journal.pone.0130662

**Published:** 2015-06-25

**Authors:** Rodolfo Nallar, Zsuzsanna Papp, Tasha Epp, Frederick A. Leighton, Seth R. Swafford, Thomas J. DeLiberto, Robert J. Dusek, Hon S. Ip, Jeffrey Hall, Yohannes Berhane, Samantha E. J. Gibbs, Catherine Soos

**Affiliations:** 1 Department of Veterinary Pathology, Western College of Veterinary Medicine, University of Saskatchewan, Saskatoon, Saskatchewan, Canada; 2 Wildlife Conservation Society, Greater Madidi-Tambopata Landscape Conservation Program, La Paz, Bolivia; 3 Environment Canada, Science & Technology Branch, Saskatoon, Saskatchewan, Canada; 4 Department of Large Animal Clinical Sciences, Western College of Veterinary Medicine, University of Saskatchewan, Saskatoon, Saskatchewan, Canada; 5 Canadian Wildlife Health Cooperative, University of Saskatchewan, Saskatoon, Saskatchewan, Canada; 6 United States Department of Interior, US Fish and Wildlife Service, Yazoo City, Mississippi, United States of America; 7 United States Department of Agriculture, Animal and Plant Health Inspection Service, Wildlife Services, National Wildlife Disease Program, Fort Collins, Colorado, United States of America; 8 United States Geological Survey, National Wildlife Health Center, 6006 Schroeder Road, Madison, Wisconsin, United States of America; 9 National Centre for Foreign Animal Disease, Canadian Food Inspection Agency, Winnipeg, Manitoba, Canada; 10 United States Fish and Wildlife Service, Division of Migratory Bird Management, Laurel, Maryland, United States of America; University of Minnesota, UNITED STATES

## Abstract

Since the spread of highly pathogenic avian influenza (HPAI) H5N1 in the eastern hemisphere, numerous surveillance programs and studies have been undertaken to detect the occurrence, distribution, or spread of avian influenza viruses (AIV) in wild bird populations worldwide. To identify demographic determinants and spatiotemporal patterns of AIV infection in long distance migratory waterfowl in North America, we fitted generalized linear models with binominal distribution to analyze results from 13,574 blue-winged teal (*Anas discors*, BWTE) sampled in 2007 to 2010 year round during AIV surveillance programs in Canada and the United States. Our analyses revealed that during late summer staging (July-August) and fall migration (September-October), hatch year (HY) birds were more likely to be infected than after hatch year (AHY) birds, however there was no difference between age categories for the remainder of the year (winter, spring migration, and breeding period), likely due to maturing immune systems and newly acquired immunity of HY birds. Probability of infection increased non-linearly with latitude, and was highest in late summer prior to fall migration when densities of birds and the proportion of susceptible HY birds in the population are highest. Birds in the Central and Mississippi flyways were more likely to be infected compared to those in the Atlantic flyway. Seasonal cycles and spatial variation of AIV infection were largely driven by the dynamics of AIV infection in HY birds, which had more prominent cycles and spatial variation in infection compared to AHY birds. Our results demonstrate demographic as well as seasonal, latitudinal and flyway trends across Canada and the US, while illustrating the importance of migratory host life cycle and age in driving cyclical patterns of prevalence.

## Introduction

Wild birds, particularly waterfowl of the order Anseriformes, are considered the natural reservoir for most subtypes of low pathogenic avian influenza viruses (AIV) [1,2]. Low pathogenic AIVs (LPAIVs) do not cause clinical signs in wild ducks; however, H5 and H7 subtypes have the potential to evolve into highly pathogenic AIV (HPAIV) when introduced into domestic bird populations [1]. HPAIVs cause large scale mortality in domestic bird populations, and some HPAIVs as well as LPAIVs may cause serious illness in humans [3–7]. In response to the emergence and spread of H5N1 HPAIV in Asia, Europe, and Africa, numerous large scale surveillance programs were initiated worldwide. Although the primary objective of most of these surveillance programs was the early detection of H5N1 HPAIV, these programs have resulted in a large amount of valuable data on LPAIVs. To our knowledge, few studies have examined large scale spatiotemporal patterns and ecological determinants of AIV infection in waterfowl at the continental scale, particularly along migratory flyways or across seasons [8]. These types of analyses are essential not only for increasing our understanding of AIV ecology in wild birds at continental levels, but also for enhancing future surveillance and response efforts, potentially identifying key locations and time periods for AIV infection risk.

In this study, we examined the role of spatiotemporal and host life cycle factors driving AIV infection in blue-winged teal (*Anas discors*; BWTE) sampled year round for multiple years over a large geographic range. Four years of data collected by AIV surveillance programs in Canada and the United States were examined using generalized linear models and a comparative modelling approach to determine the effects of year, season (as defined by stage of annual cycle of the host), flyway, latitude, and demographic factors (age and sex) on the probability of AIV infection in blue-winged teal. This species provides an ideal model for studying determinants of AIV infection in waterfowl, because it has the largest migratory range among dabbling ducks and individuals are highly gregarious [9], and are commonly infected with LPAI viruses [10–12].

Previous studies in waterfowl have detected high prevalences of AIV in hatch year (HY) birds prior to fall migration when large numbers of birds aggregate, favoring transmission of virus through the oral-fecal route [1,10,12–19] with decreasing prevalences as birds migrate further south [20,21]. In our models, we explored whether similar seasonal and latitudinal trends would be observed in BWTE, over a larger geographic distribution compared to previous studies conducted in North America. We investigated whether host life cycle and age were driving factors in previously observed seasonal patterns examining interactions between age and season. We hypothesized that there would be minimal to no seasonal differences in AIV infection for AHY birds, but that for HY birds, the effect of age would decrease during the course of the first year of life as they develop immunity, and become less likely to be infected with time. We also explored longitudinal trends, using flyway as a covariate, and expected that flyways with the highest densities of BWTE would have higher prevalences of infection. Sex was also included in our models as previous studies have shown males to be more susceptible to infection compared to females [10,19,22], although this trend has not been consistent among studies [15,18,23].

Thus, in this study our goals were to evaluate the role of key spatiotemporal and host life cycle factors driving AIV infection in a migratory host over a large geographic range.

## Materials and Methods

**Fig 1:**
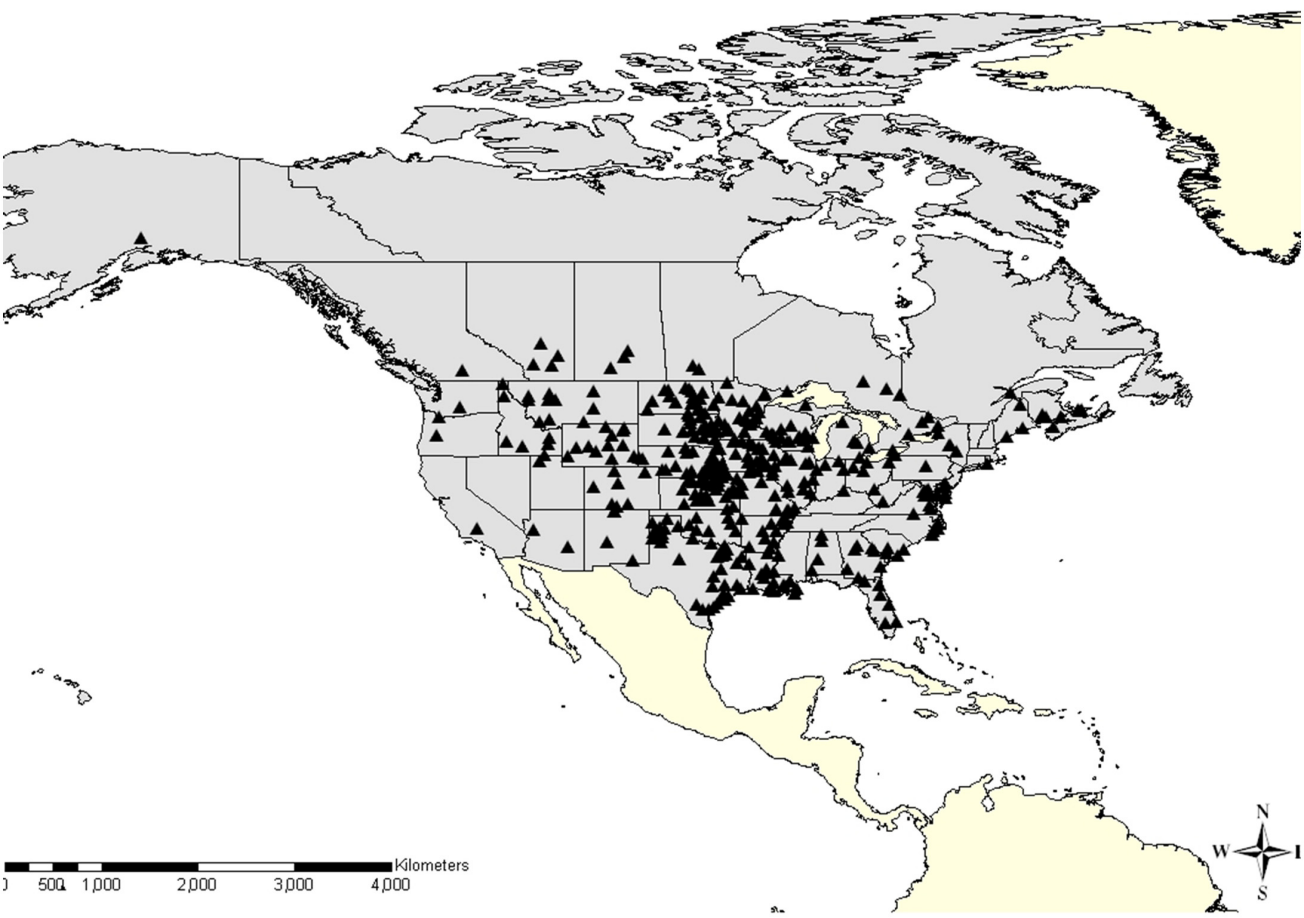
Locations of capture sites of Blue-winged Teal sampled for low pathogenic avian influenza virus across Canada and the United States, from 2007 to 2010.

Datasets were obtained courtesy of Canada’s Inter-agency Wild Bird Influenza Survey (2007–10), the US plan “An Early Detection System for Asian H5N1 Highly Pathogenic Avian Influenza in Wild Migratory Birds (US Department of Agriculture and the US Department of Interior, 2007–10), [10,24,25] and studies conducted by the NIH Centers of Excellence for Influenza Research and Surveillance. Sampling of BWTE was distributed across 42 states in the US and 9 provinces in Canada (Fig 1). For each bird, information on location, date, band number, age and sex were recorded. Aging protocols were consistent across agencies [26]. Combined cloacal and oropharyngeal swabs in all surveys were analyzed for the presence of the matrix protein gene segment common to all influenza A viruses using the real-time reverse transcriptase polymerase chain reaction (RRT-PCR) assay described by Spackman *et al*. [27], using standardized methods across all labs. A sample with a threshold cycle value of 35 and below was considered positive. Datasets included PCR results for individual birds tested, along with the full complement of field data containing all variables listed in Table 1.

**Table 1:** List of outcome and explanatory variables used in models examining demographic and spatiotemporal determinants of low pathogenic avian influenza virus infection in Blue-winged Teal in Canada and the United States, 2007–10.

Variable	Type	Definition	Description
AIV infection	Categorical, outcome variable	Positive suggests current infection based on RRT-PCR	Binomial outcome–positive or negative
Sex	Categorical, explanatory	Gender of bird	Female or Male
Age	Categorical, explanatory	Age of bird at capture	Hatch year (<1 year old, HY and SY) or after hatch year (>1 year old, AHY and ASY)
Year	Categorical, explanatory	Year of sampling	2007–2010
Flyway	Categorical, explanatory	Migratory flyway where sampling occurred [19]	Atlantic, Mississippi, Central, or Pacific
Latitude	Continuous, explanatory	Latitude of sampling location	Decimal degrees, standardized
Blue-winged teal stage of annual life cycle–“Season”	Categorical, explanatory	Stage of life cycle of BWTE [18]	July-August: Staging, End of Moult, Preparation for fall migration; Sept-Oct: Fall migration; Nov-June: Wintering, Spring migration, breeding period

### Statistical analyses

Descriptive analyses were conducted in Microsoft Excel, and inferential statistical analyses were performed in R [28]. Records missing any field or laboratory data were excluded from analysis. To investigate determinants of AIV infection, we analyzed our data using generalized linear models with a binominal response variable, where AIV positive = 1 and AIV negative = 0, based on maximum-likelihood approximation. Explanatory variables are described in Table 1. To examine temporal trends of AIV infection in BWTE, season categories were created from the annual life cycle of BWTE: July-August (staging prior to fall migration), September-October (fall migration), and November-June (a pooled category created due to small sample sizes, including wintering stage and spring migration (November-April), and breeding period (May-June)) [9]. Migratory flyways were classified using the four administrative categories based on waterfowl migration corridors: Pacific, Central, Mississippi, and Atlantic flyway [29]. The assumption of linearity for the continuous variable “latitude” was examined using two methods: categorization and inclusion of a quadratic term. Both methods revealed a similar non-linear association, thus we included the quadratic term in our final model set. Latitude was standardized to have a mean of 0 and a SD of 1 before including in models and before creating the quadratic term. For simplicity, and to allow us to examine the interaction between age and season, we classified age into two categories: hatch year (HY) and after hatch year (AHY). In our analyses, HY birds included all birds that were less than one year of age (including second year (SY) birds sampled between January and June), and AHY included all birds that were greater than one year of age (including after second year birds (ASY) sampled between January and June.

To explain variation in AIV status, a set of models was built based on general guidelines [30,31] (S1 Table). First we explored simple models including only one predictor. All variables, except for Sex, improved the null model (lowered AICc) therefore we combined them in biologically meaningful ways in more complex models, while minimizing the number of models explored. The continuous variable "Latitude" was assessed for linearity with the outcome. We included Sex in more complex models based on its hypothesized importance as a basic demographic variable. We then added year and flyway followed by latitude and quadratic latitude to capture the non-linear nature of the association with the outcome. We included biologically meaningful interactions such as Age*Season and Age*Sex if these improved AICc in simpler models.

Model selection was carried out using the Akaike information criterion corrected for small sample size (AIC_c_; [30]) to rank competing models. We considered the model with the lowest AIC_c_ to have the best support given the data. The best supported model passed the Pearson χ^2^ goodness of fit test.

To create maps illustrating probability of AIV infection, predicted probability of AIV infection for each individual was calculated based on the best supported model. These values were group-averaged across sexes and years for each sampling location, and then were interpolated with the spatial analysis tool in ArcGIS [32], using Natural Neighbor interpolation procedures to obtain values for unsampled locations based on known surface values of adjacent sites [33].

## Results

### Descriptive statistics

Records of 13,574 BWTE tested for AIV infection in Canada and the US from 2007 to 2010 were obtained. Apparent prevalence of AIV infection was 17.8% overall, 18.2% (95% confidence interval (CI) = 16.8, 19.6) in Canada (n = 2,989), and 17.8% (95% CI = 16.8, 19.6) in the US (n = 10,585). Variation in apparent prevalence of AIV was observed by age, season, year and flyway (Table 2).

**Table 2:** Apparent prevalence of low pathogenic avian influenza virus in Blue-winged Teal (*Anas discors*) in Canada and the United States, 2007–2010.

Proportion	n	% AIV positive	95% CI
Overall	13574	17.8	17.17, 18.45
By age			
HY	9113	21.4	20.57, 22.25
AHY	4461	10.4	9.59, 11.38
By sex			
Female	7491	17.6	16.78, 18.51
Male	6083	18.0	17.05, 18.99
By season			
Nov-June	1463	9.5	8.12, 11.06
July-Aug	3395	22.9	21.51, 24.38
Sept-Oct	8716	19.3	18.49, 20.15
By year			
2007	3347	19.21	17.91, 20.58
2008	3636	12.24	11.21, 13.35
2009	3085	18.41	17.08, 19.82
2010	3506	21.68	20.35, 23.07
By flyway (ref = Atlantic)			
Pacific	40	7.5	2.6, 19.8
Central	7311	20.8	19.87, 21.73
Mississippi	4934	15.6	14.62, 16.65
Atlantic	1289	9.6	8.13, 11.35

Abbreviations: HY = hatch year (including SY), AHY = after hatch year (including ASY), AIV = Avian influenza virus matrix protein gene.

### Modelling the ecology of low pathogenic AIV infection in blue-winged teal

**Fig 2:**
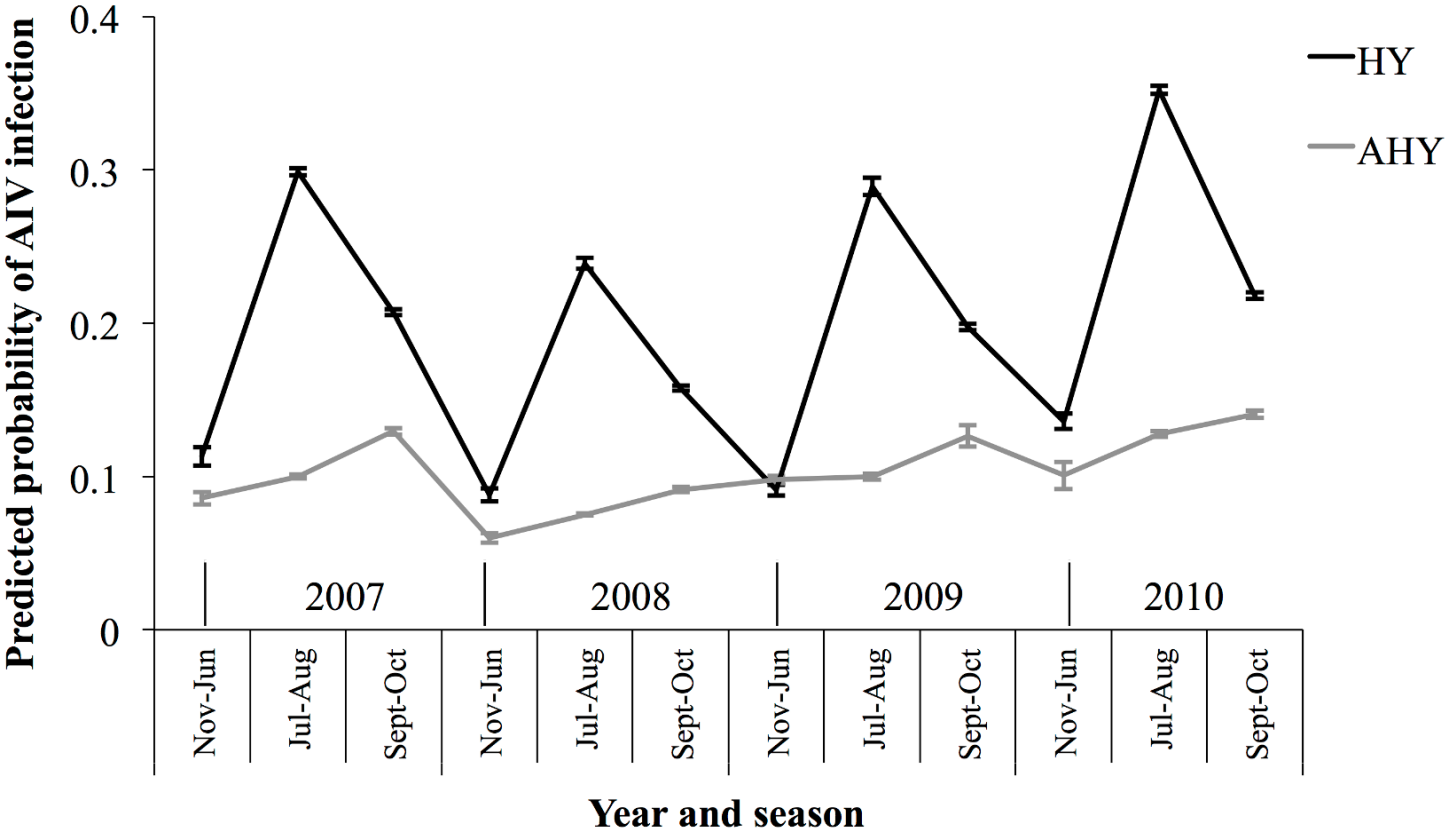
Annual and seasonal means of predicted probability of avian influenza virus infection in Blue-winged Teal in Canada and the US from 2007–2010. Point predictions were based on the best-supported model (Table 3) and were averaged across both sexes, and all flyways and latitudes of data. Confidence intervals are calculated based on the variance around the group-mean within each year-season-age category. Hatch year (HY) and after hatch year (AHY) age groups shown separately.

**Fig 3:**
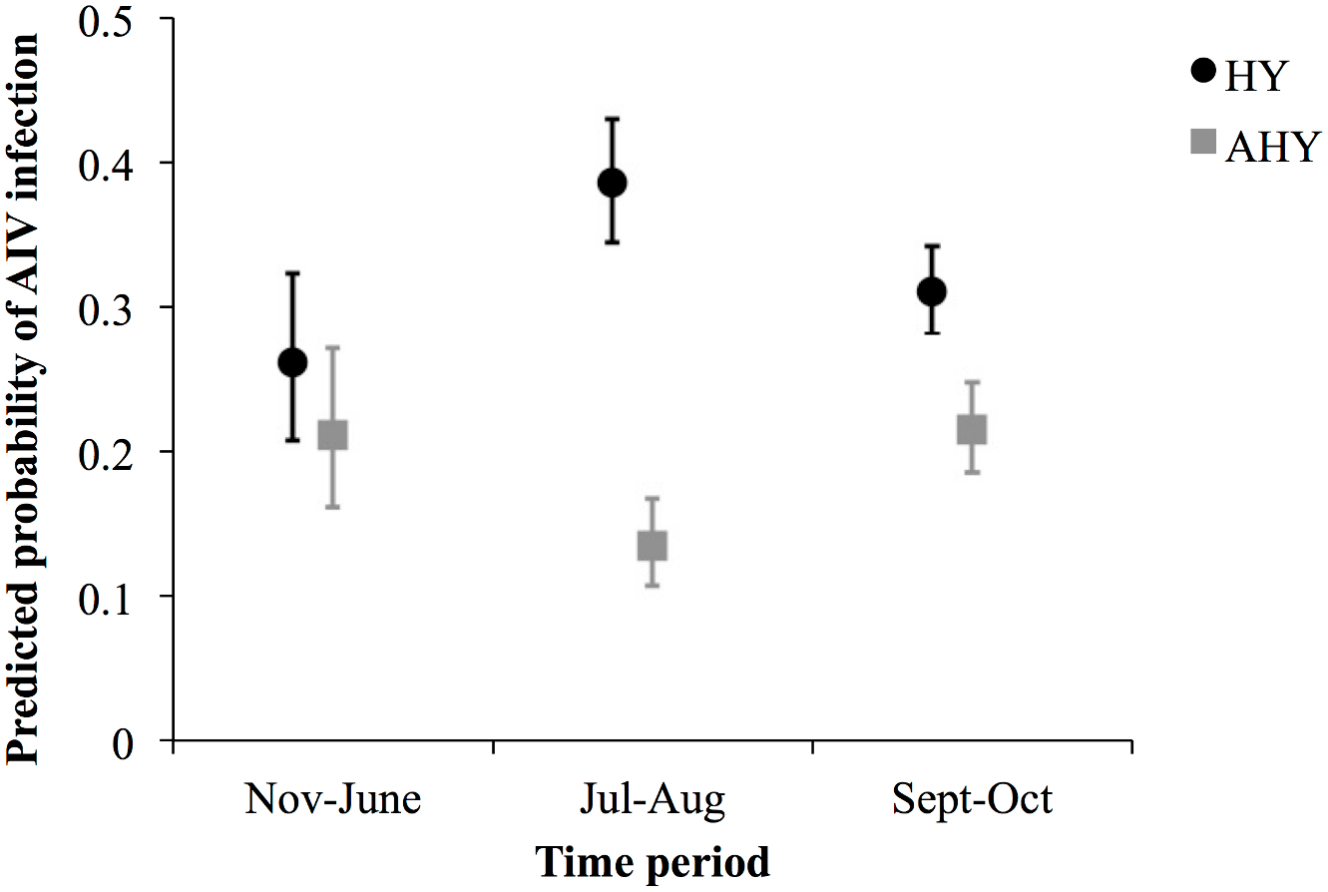
Predicted probability of avian influenza virus (AIV) infection in Blue-winged Teal, illustrating the interaction between age and season. Predictions are based on the best-supported model (Table 3) with other explanatory variables set at male (Sex), 2010 (Year), central flyway (Flyway) and mean latitude. Hatch year: HY, after hatch year: AHY. Error bars represent 95% confidence intervals, based on SE calculated by the delta method.

Results of all examined models are presented in S1 Table. In our best-supported model (Table 3), there was an interaction between age and stage of annual cycle. HY birds were more likely to be positive for AIV compared to AHY birds in July-August just prior to fall migration (odds ratio, OR = 4.06, 95% CI = 3.29, 5.03; Figs 2 and 3). This effect decreased in September-October (fall migration), and was absent in November-June (winter, spring migration, breeding) (OR = 1.65, 95% CI = 1.43, 1.91 and OR = 1.32, 95% CI = 0.92, 1.89, respectively; Fig 3). In HY birds, the probability of being infected with AIV was highest in July-August (OR = 1.78, 95% CI = 1.31, 2.42, compared to Nov-June) and declined during fall migration (September-October; OR = 0.72, 95% CI = 0.62, 0.83, compared to Jul-Aug; Figs 2 and 3). A different seasonal pattern was observed in AHY BWTE which, unlike the HY birds, had the lowest probability of infection in July-August (OR = 0.58, 95% CI = 0.40, 0.84, compared to Nov-June), with no difference between Sept-Oct and Nov-June (Table 3, Fig 3). Males were 23% more likely to be infected compared to females (OR = 1.23, 95% CI = 1.11, 1.35).

**Table 3:** Best-supported logistic regression model fitted to explain variation in AIV infection probability in Blue-winged Teal in Canada and the US, 2007–2010 (n = 13,574).

Variables	β	SE	95% CI
Intercept	-1.659	0.134	-1.922, -1.395
Age (ref = HY)	-1.402	0.109	-1.615, 1.190
Sex (ref = Female)	0.206	0.050	0.108, 0.303
Season (ref = July-August)			
Sept-Oct	-0.334	0.077	-0.485,0.183
Nov-June	-0.576	0.157	-0.883, 0.269
Year (ref = 2007)			
2008	-0.306	0.069	-0.442, 0.170
2009	0.032	0.067	-0.100, 0.164
2010	0.288	0.064	0.162, 0.414
Latitude	0.157	0.037	0.085, 0.229
(Latitude)2	-0.097	0.030	-0.156, 0.038
Flyway (ref = Atlantic)			
Pacific	-0.410	0.615	-1.613, 0.798
Central	0.702	0.105	0.496, 0.908
Mississippi	0.440	0.107	0.231, 0.650
Age*Season			
HY- Sept-Oct	0.903	0.131	0.646, 1.160
HY- Nov-June	1.125	0.214	0.705, 1.544

Abbreviations: *β* = coefficient estimate, SE = standard error, CI (95% confidence interval), ref = reference, HY = hatch year, AHY = after hatch year. Latitude is standardized to have a mean of 0 and standard deviation of 1. The quadratic term is the standardized latitude squared. Age*Season is the interaction term.

Year was an informative variable, with highest risk of infection in 2007 and 2010 (Table 3, Fig 2). Migratory flyway was a strong predictor of AIV infection. Blue-winged teal were more likely to test positive for AIV in the Central and Mississippi flyways compared to birds in the Atlantic flyway (Table 3) (OR = 2.02, 95% CI = 1.64, 2.48, OR = 1.55, 95% CI = 1.26, 1.92, respectively). Birds in the Central flyway were more likely to be positive for AIV than those in the Mississippi flyway (OR = 1.3, 95% CI = 1.17, 1.44). AIV prevalence in BWTE in the Pacific flyway was not statistically different from that in other flyways, however the small sample size in the Pacific flyway (n = 40) does not allow us to make strong conclusions from this result.

**Fig 4:**
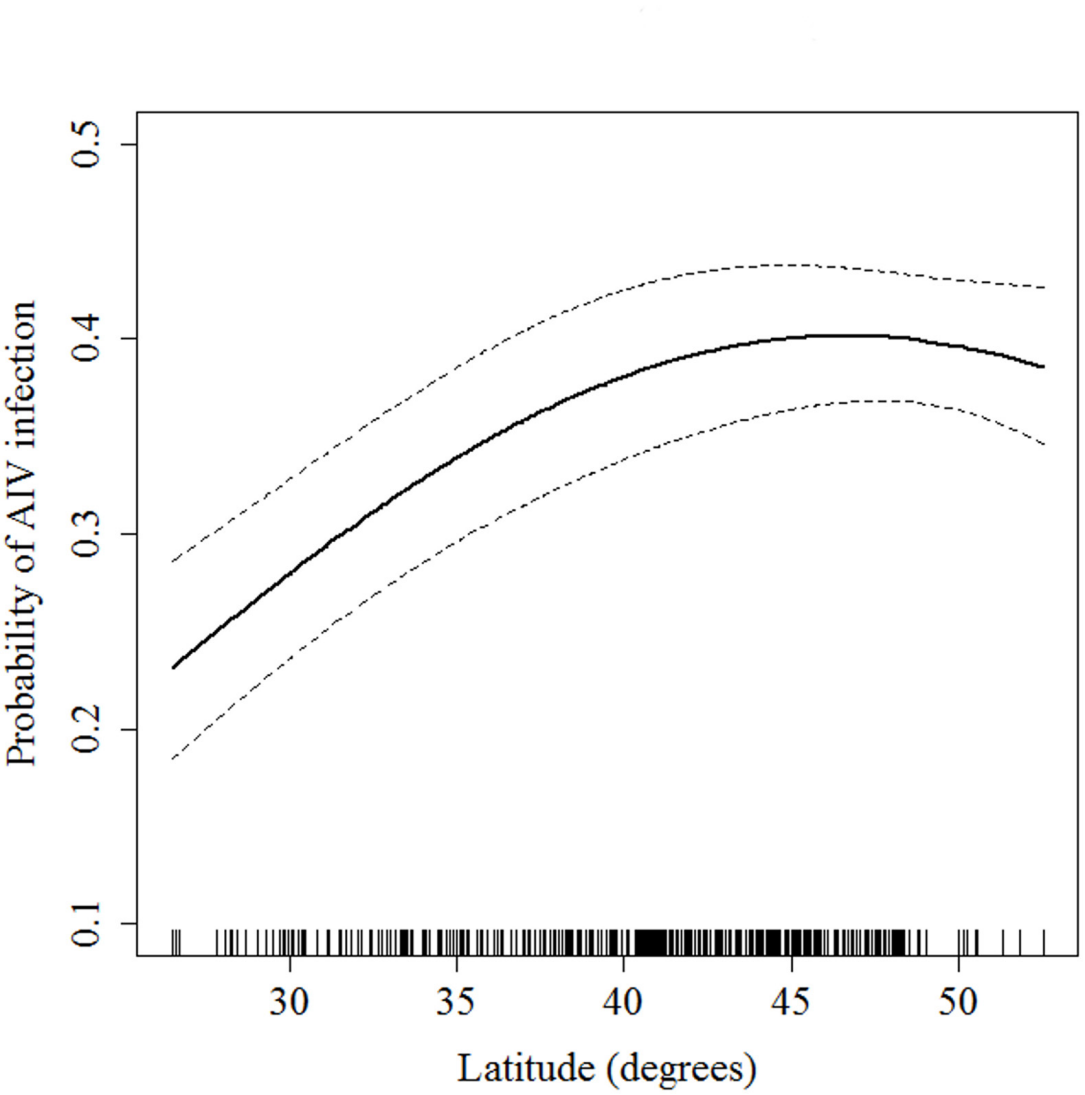
Probability of avian influenza virus infection in Blue-winged Teal as a function of latitude of sampling location. Predicted probability was calculated based on the best-supported model (Table 3) with Age, Sex, Flyway, Season and Year set at hatch year, male, central, July-August and 2010 categories, respectively. Error bars represent 95% confidence intervals.

**Fig 5:**
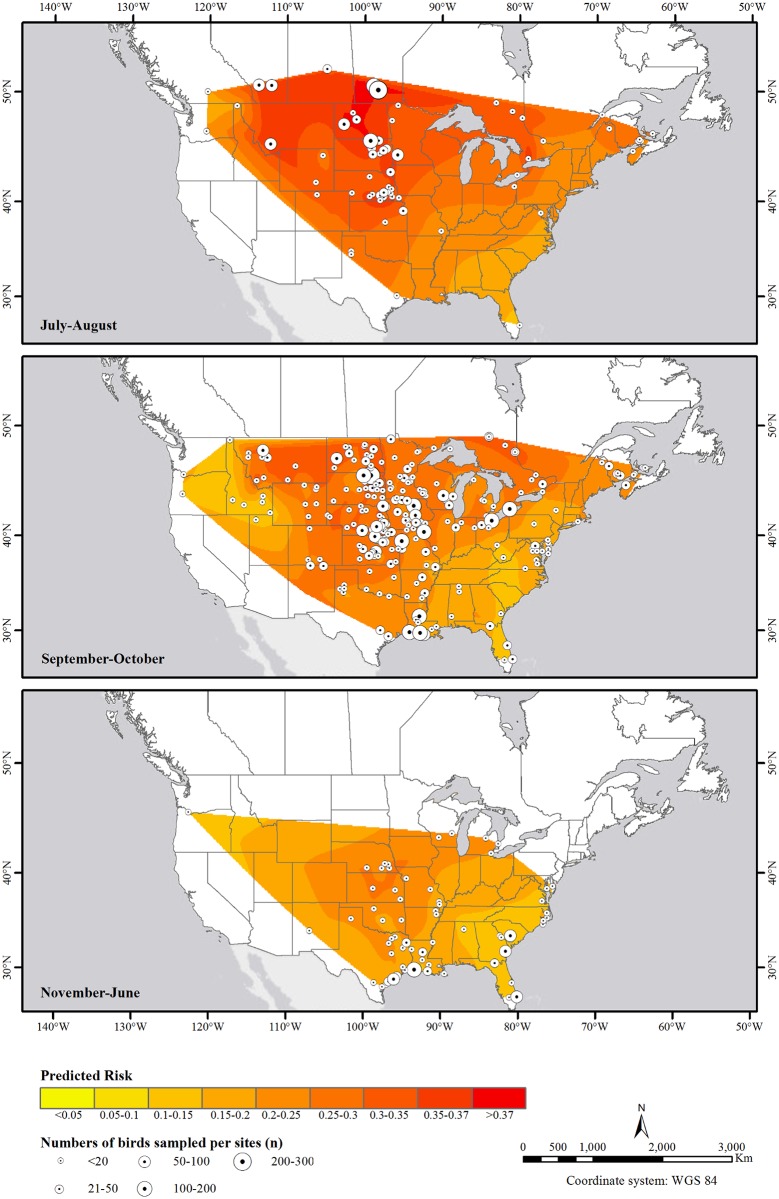
Predicted risk of avian influenza virus infection in hatch year Blue-winged Teal in Canada and the US from 2007–2010 at different stages of the annual life cycle. Natural neighbor interpolation spatial analysis was applied to predicted probability values calculated for all hatch year birds based on the best-supported model (Table 3), and averaged across all years and both sexes for each sampling site (circles).

**Fig 6:**
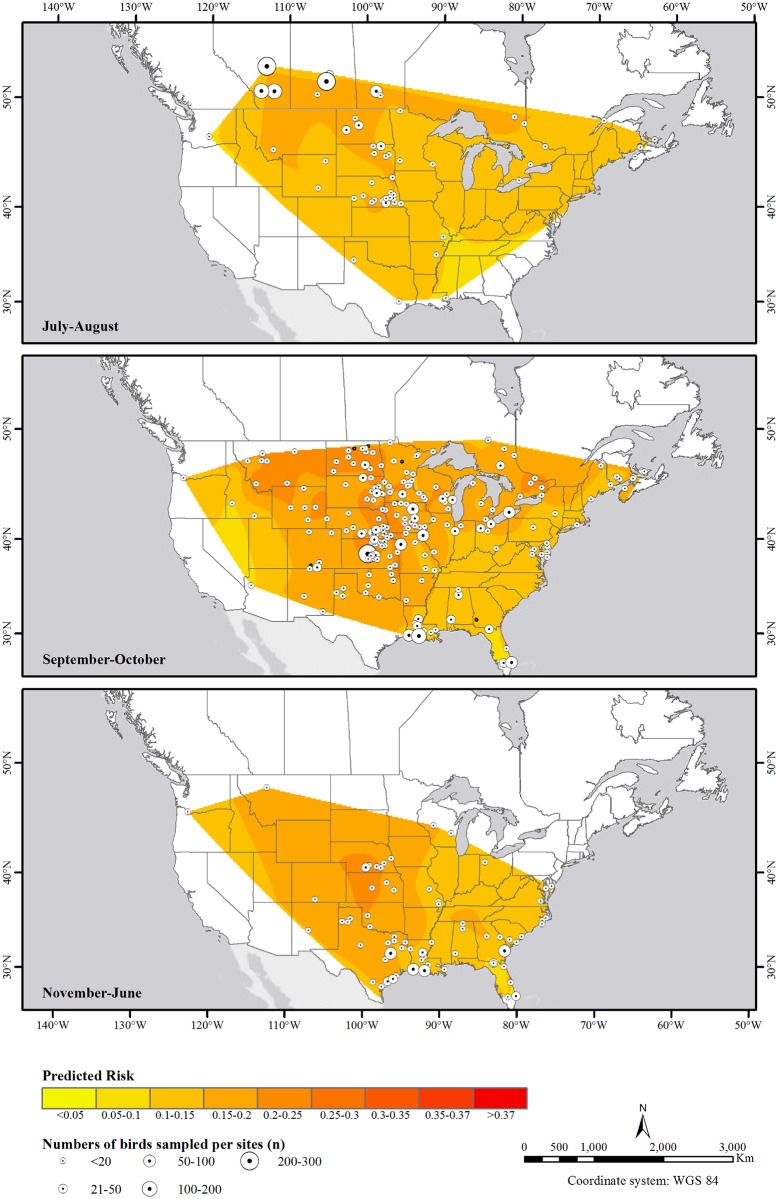
Predicted risk of avian influenza virus infection in after hatch year Blue-winged Teal in Canada and the US from 2007–2010 at different stages of the annual life cycle. Natural neighbor interpolation spatial analysis was applied to predicted probability values calculated for all after hatch year birds based on the best-supported model (Table 3), and averaged across all years and both sexes for each sampling site (circles).

The probability of AIV infection was positively associated with latitude up to about 43° north, after which there was no further increase in risk of infection with latitude, and a slight declining trend beyond ~48° north (for HY birds in July-August, Table 3, Fig 4). The seasonal and spatial trends discussed above are further illustrated in Figs 5 and 6, which show the spatial distribution of predicted probability of AIV infection in HY and AHY birds, respectively. Whereas latitudinal, seasonal and flyway trends appear to be very prominent in HY birds, these trends are much less apparent in AHY birds which not only have lower apparent prevalences, but also have much less spatiotemporal variation in AIV infection probability.

## Discussion

Using data collected during surveillance programs in Canada and the United States from 2007 to 2010, we identified demographic as well as seasonal, latitudinal, flyway, and annual trends in AIV infection in BWTE across a large geographic range in North America, while illustrating the importance of migratory host annual cycle and age in driving seasonal cycles in prevalence.

A unique finding of our study was an interaction between age and season (as defined by stage of annual cycle of BWTE). Previous studies have shown that HY waterfowl are more likely to be infected with AIV than adults [10,22,34]. However our results also indicated that while HY BWTE are more likely to be infected with AIV during July to October (staging and fall migration), they were no more likely to be infected compared to AHY birds by the November-June season (wintering stage) likely due to newly acquired immunity to AIV.

Seasonal cycles of AIV infection appeared to be driven by the dynamics of AIV infection in HY birds which had more prominent temporal as well as spatial variation in AIV prevalence compared to AHY birds (Figs 2, and 5 vs 6). Juveniles were also found to be major drivers for seasonal epizootics in mallards [35]. HY birds had the highest estimated prevalences in July-August compared to other stages in the annual cycle as that is when they are most vulnerable to infection, having no previously acquired immunity. Not only is July-August the period with the expected highest HY:AHY ratio in the population, it is also the period with the highest population densities and mixing of waterfowl of different species from numerous locations [9], and thus, it is likely the period with the highest contact rates for transmission between infected and susceptible individuals. Interestingly, AHY BWTE were least likely to be infected in July-August and more likely to be infected in Sept-through June. It is possible that, at the time of sampling, AHY birds had already developed immunity to viruses circulating in July-August, and were more vulnerable to novel strains encountered during the subsequent stages in the annual cycle. The decreasing overall trend as birds migrate south for wintering would likely be due to the increased proportion of HY birds becoming immune, resulting in reduced rates of transmission.

Similar to other studies, we found that male BWTE were more likely to be infected with AIV compared to females [10,19], suggesting an innate difference in their vulnerability to infection possibly due to physiological (e.g., hormonal) and/or behavioural (e.g., foraging, aggression, gregariousness) differences [36].

Latitude may be a complex variable in our models, important partially through its association with season, which drives the movement of birds northward and southward, but also because of changes in environmental or climatic factors with latitude. Environmental temperatures are cooler and more variable with increasing latitudes. AIVs have been shown to persist for long periods at cooler temperatures [37], with cold temperatures increasing the potential for viruses to survive overwinter [38]. The positive association between AIV infection and latitude was not linear, with no additional increased risk in AIV infection beyond ~43° north, beyond which there was a plateau and slightly declining trend. Although colder temperatures are generally better for virus survival, it has been demonstrated experimentally that extremely cold temperatures (below -30°C) may decrease virus survival in the environment [39], which may explain this non-linear trend. In addition, latitudinal patterns were most prominent in July-August and during fall migration, particularly in HY birds, thus the latitudinal trend may have been driven in part by the interaction between age and host annual life cycle (season) and associated with changes in host density and increases in immunity as birds disperse among wintering areas southward.

BWTE in the Central flyway were more likely to be infected with AIV than birds in the Mississippi flyway, and birds sampled in either of these flyways were more likely to be infected than those in the Atlantic flyway. This pattern is different from that in a study of several species of migratory waterfowl [40] and may be a reflection of BWTE population density. The highest density of breeding blue-winged teal occurs in the central prairies of the US and Canada, spanning southeastern SK, southwestern MB, and the Dakotas which encompass the most important breeding areas for this species [41].

Few studies have examined large scale spatiotemporal patterns and ecological determinants of AIV infection in waterfowl at the continental scale and across seasons. Our results not only provide further evidence for the role of demographic and spatiotemporal factors such as latitude, flyway, and season in AIV infection, but also illustrates the importance and interaction of host migratory ecology and age in driving seasonal and geographic patterns of prevalence. Future analyses of spatio-temporal patterns in AIV infection for migratory species may be improved by using an inter-continental approach that incorporates more data from wintering areas further south, particularly for species like BWTE which have wintering ranges extending into Central and South America.

This study enhances our knowledge of the ecology of low pathogenic AIVs in wild migratory waterfowl, and provides key information that can be used to enhance future surveillance and response efforts, potentially identifying key locations and time periods for AIV infection risk for this particular species. This information is particularly relevant given the recent detection of HPAIVs of Eurasian lineage (H5N8, H5N2) in wild birds in North America for the first time [42,43], and the renewed interest in live bird AIV surveillance programs in Canada and the US.

## Supporting Information

S1 Table Models fitted to explain variation in AIV infection probability in blue-winged teal sampled in the US and Canada as part of national surveillance programs from 2007 to 2010 (n = 13,574). (DOCX)

S2 Table Number of Blue-winged Teal (*Anas discors*) sampled and testing positive for low pathogenic avian influenza virus in Canada and the United States, 2007–2010, by month. (DOCX)
