# Supplementary material for: Demographic and Spatiotemporal Patterns of Avian Influenza Infection at the Continental Scale, and in Relation to Annual Life Cycle of a Migratory Host
Source: PLoS One. 2015 Jun 25;10(6):e0130662. doi: 10.1371/journal.pone.0130662 (PMC4481355; doi:10.1371/journal.pone.0130662)
Supplement: S1 Table — (DOCX) [file pone.0130662.s001.docx]

# Supporting information

S1 Table. Models fitted to explain variation in AIV infection probability in Blue-winged Teal sampled in the US and Canada as part of national surveillance programs from 2007 to 2010 (n = 13,574).

| Model | K | AIC_c_ | ΔAIC_c_ | ω_i_ |
| --- | --- | --- | --- | --- |
| Age + Sex + Age*Season + Season + Year + Flyway + Latitude + (Latitude)^2^ | 15 | 12115.28 | 0 | 0.999 |
| Age + Sex + Age*Sex + Season + Year + Flyway + Latitude + (Latitude)^2^ | 14 | 12151.80 | 36.52 | 0.000 |
| Age + Sex + Season + Year + Flyway + Latitude + (Latitude)^2^ | 13 | 12167.97 | 52.69 | 0.000 |
|  |  |  |  |  |
| Age + Sex + Season + Year + Flyway + Latitude | 12 | 12186.08 | 70.80 | 0.000 |
| Age + Sex + Season + Year + Flyway | 11 | 12204.54 | 89.26 | 0.000 |
| Age + Sex + Season + Year | 8 | 12265.76 | 150.48 | 0.000 |
| Age + Sex + Season + Age*Season | 7 | 12275.05 | 159.77 | 0.000 |
| Age + Sex + Season | 5 | 12325.26 | 209.98 | 0.000 |
| Age + Sex + Age*Sex | 4 | 12443.88 | 328.60 | 0.000 |
| Age + Sex | 3 | 12452.14 | 336.86 | 0.000 |
| Age | 2 | 12453.49 | 338.20 | 0.000 |
| Latitude + (Latitude)^2^ | 3 | 12536.33 | 421.04 | 0.000 |
| Latitude | 2 | 12577.62 | 462.33 | 0.000 |
| Season | 3 | 12584.52 | 469.24 | 0.000 |
| Flyway | 4 | 12590.89 | 475.61 | 0.000 |
| Year | 4 | 12598.33 | 483.05 | 0.000 |
| Null | 1 | 12716.16 | 600.88 | 0.000 |
| Sex | 2 | 12717.86 | 602.58 | 0.000 |
