# Supplementary material for: Demographic and Spatiotemporal Patterns of Avian Influenza Infection at the Continental Scale, and in Relation to Annual Life Cycle of a Migratory Host
Source: PLoS One. 2015 Jun 25;10(6):e0130662. doi: 10.1371/journal.pone.0130662 (PMC4481355; doi:10.1371/journal.pone.0130662)
Supplement: S2 Table — (DOCX) [file pone.0130662.s002.docx]

# Supporting information

S2 Table. Number of Blue-winged Teal (*Anas discors*) sampled and testing positive for low pathogenic avian influenza virus in Canada and the United States, 2007-2010, by month.

|  |  | |  | |  |  | |  | |  |
| --- | --- | --- | --- | --- | --- | --- | --- | --- | --- | --- |
|  | # AIV pos/# sampled | | | | | | | | |  |
|  |  |  | |  | | |  | |  | |
|  | HY | HY | | AHY | | | AHY | | Overall | |
|  | F | M | | F | | | M | |  | |
| Month |  |  | |  | | |  | |  | |
| 1 | 3/40 | 7/45 | | 3/43 | | | 3/64 | | 16/192 | |
| 2 | 3/8 | 4/25 | | 4/28 | | | 4/41 | | 15/102 | |
| 3 | 6/45 | 9/86 | | 3/31 | | | 8/91 | | 26/253 | |
| 4 | 0/13 | 1/4 | | 0/27 | | | 0/11 | | 1/55 | |
| 5 | 0/2 | -- | | 0/10 | | | 0/3 | | 0/15 | |
| 6 | -- | -- | | 0/3 | | | 0/1 | | 0/4 | |
| 7 | 4/27 | 3/26 | | 1/6 | | | 0/6 | | 8/65 | |
| 8 | 343/1139 | 290/878 | | 35/363 | | | 93/950 | | 761/3330 | |
| 9 | 557/2984 | 411/2032 | | 160/1249 | | | 79/817 | | 1207/7082 | |
| 10 | 128/739 | 134/537 | | 28/246 | | | 13/112 | | 303/1634 | |
| 11 | 23/218 | 14/141 | | 14/176 | | | 15/143 | | 66/678 | |
| 12 | 3/67 | 7/57 | | 3/27 | | | 0/13 | | 13/164 | |
| Total | 1070/5282 | 880/3831 | | 251/2209 | | | 215/2252 | | 2416/13574 | |

Abbreviations: HY = hatch year (including SY), AHY = after hatch year (including ASY), AIV = Avian influenza virus matrix protein gene., pos= positive, neg=negative, F=female, M=male
